# Philadelphia Hospital, Blockley

**Published:** 1858-09

**Authors:** 


PHILADELPHIA HOSPITAL, BLOCKLEY.—At the recent election for Chief Resident-Physician of this institution, Dr. McClintock was removed and Dr. R. K. Smith was appointed in his stead. No change has been made in the corps of Assistant Resident-Physicians. We speak advisedly when we say they are all rejoiced at getting rid of their late *chief*.

